# Smartphone-enabled optofluidic exosome diagnostic for concussion recovery

**DOI:** 10.1038/srep31215

**Published:** 2016-08-08

**Authors:** Jina Ko, Matthew A. Hemphill, David Gabrieli, Leon Wu, Venkata Yelleswarapu, Gladys Lawrence, Wesley Pennycooke, Anup Singh, Dave F. Meaney, David Issadore

**Affiliations:** 1 Department of Bioengineering, School of Engineering and Applied Sciences, University of Pennsylvania. Philadelphia, Pennsylvania, United States; 2 Department of Electrical and Systems Engineering, School of Engineering and Applied Sciences, University of Pennsylvania. Philadelphia, Pennsylvania, United States; 3 Department of Neurosurgery, Perelman School of Medicine, University of Pennsylvania, Philadelphia, Pennsylvania, United States

## Abstract

A major impediment to improving the treatment of concussion is our current inability to identify patients that will experience persistent problems after the injury. Recently, brain-derived exosomes, which cross the blood-brain barrier and circulate following injury, have shown great potential as a noninvasive biomarker of brain recovery. However, clinical use of exosomes has been constrained by their small size (30–100 nm) and the extensive sample preparation (>24 hr) needed for traditional exosome measurements. To address these challenges, we developed a smartphone-enabled optofluidic platform to measure brain-derived exosomes. Sample-to-answer on our chip is 1 hour, 10x faster than conventional techniques. The key innovation is an optofluidic device that can detect enzyme amplified exosome biomarkers, and is read out using a smartphone camera. Using this approach, we detected and profiled GluR2+ exosomes in the post-injury state using both *in vitro* and murine models of concussion.

## Introduction

Mild traumatic brain injury (mTBI), i.e. concussion, occurs in 1.7–2 million people each year^1^,^2^. Most mTBI patients recover within one year following the incident, but an estimated 10% of mild cases result in a long-term disability including seizures, and emotional and behavioral issues^3^. Moreover, repetitive mTBI is now linked to long-term neurodegenerative changes in the brain^4^,^5^,^6^. However, clinical tools to examine the biochemical and genetic changes following mTBI are lacking^5^,^7^. Without access to detailed, individual-based prognostic markers to evaluate recovery, diagnostics for prospectively identifying patients with postconcussion syndrome are limited to monitoring patients for symptoms that may appear weeks to months following an injury.

Although there is a great interest in developing biomarkers for both the detection and treatment of concussion, the molecular biomarkers are difficult to detect for clinical use in mTBI because the blood-brain barrier (BBB) remains intact after injury^7^,^8^,^9^. As such, previous work has focused mainly on detecting markers in the cerebral spinal fluid (CSF), which is in direct contact with the extracellular space within the brain. There has been great interest in blood-borne markers, which can be collected more easily than CSF. However, the use of biomarkers from whole blood has been limited by multiple factors including extremely low concentration (fM-pM), proteolytic degradation, clearance by the liver or kidney, and binding of potential biomarkers to carrier proteins^7^.

**Figure 1:**
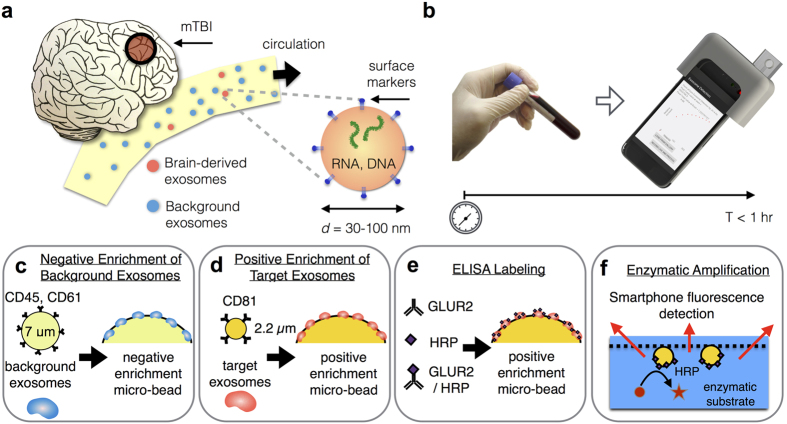
Rapid exosome-based prognosis of mild traumatic brain injury (mTBI) on a microchip. (**a**) We developed a microfluidic-based platform to isolate and profile brain-derived exosomes to diagnose mild traumatic brain injury (mTBI). Following mTBI, brain-derived exosomes (pink) that carry molecular information from their mother cells circulate in the blood amongst a vast background exosomes (blue). (**b**) The results, from serum to a digital read out, are automated on the microchip platform and can be carried out in less than 1 hour, empowering healthcare providers with real-time molecular information at the point of medical care. (**c**) On our microchip, first CD45 and CD61 coated functionalized microbeads are used to negatively select exosomes from abundant cell types (leukocytes, platelets) prior to positive selection to diminish background, which would otherwise limit sensitivity. (**d**) CD81 (a pan-exosome marker) functionalized microbeads are then used to capture the remaining exosomes. (**e**) These isolated exosomes are labeled with GluR2 antibodies, functionalized with the catalyst Horseradish Peroxidase (HRP). (**f**) The presence of HRP on these microbeads, leads to the enzymatic generation of fluorescent dye, which can be easily detected by our smartphone based fluorescence detection.

Recently, exosomes, small (30–100 nm diameter) membrane-bound vesicles carrying proteins, RNA, and DNA from their mother cells have been found to pass through the BBB, offering a new opportunity to evaluate cell-based changes in neurons and glia behind the barrier after trauma^9^,^10^,^11^,^12^ (Fig. 1a). An increase in circulating exosomes appears in the blood of traumatically brain injured patients following injury^10^,^11^. However, clinical use of exosomes has been limited due to the small size of exosomes (~30–100 nm) and the extensive sample preparation (>24 hr) needed for traditional exosome measurements. The scarcity of brain-derived exosomes relative to those originating from other major organs and hematopoietic cells in the circulation, their overlap in size with other nanoscale objects (*e.g.* cell debris) present in clinical samples, the short half-life of protein surface markers on the exosomes once removed from the body, and limited recovery rate of traditional exosome isolation methods further complicate these measurements^11^.

To address these challenges, we developed our microfluidic-based Mobile Exosome Detector (μMED) to quickly isolate and profile (<1 hr) brain-derived exosomes (Fig. 1b). This self-contained diagnostic, which is read out using only a smartphone camera, is designed to empower physicians with real-time molecular measurements of the recovering brain. Compared to conventional exosome analysis techniques that require more than a day to complete^13^, μMED requires <1 hour, is automated, and is practical for routine clinical use. The key innovations that make this work possible are 1. We combine both negative and positive microbead-based immunocapture of exosomes onto our chip. Negative selection depletes the vast majority of exosomes that are not brain-derived, enabling positive isolation and profiling of exosomes with high specificity and sensitivity. Furthermore, our on-chip capture scheme enables rapid (<1 hr) isolation of brain-derived exosomes without the need of bulky equipment (*e.g.* ultracentrifuge). Moreover, whereas ultracentrifugation isolates exosomes based on size, our method specifically captures exosomes based on their expression of specific surface markers, thus reducing copurification. 2. We use on-chip enzyme amplification to convert the very weak signal from the exosomes into an easily measured fluorescence signal. As such, μMED can detect exosomes using only a smartphone camera and off-of-the shelf optical components packaged into a low-cost 3D printed device. The modularity of the μMED device allows a wide variety of surface markers on the isolated exosomes to be profiled. In this work, we focus our attention on the the AMPA receptor subunit, glutamate receptor 2 (GluR2). GluR2 plays important roles in synaptic function and plasticity and its changes in trauma, ischemia, and epilepsy are well described^14^,^15^,^16^,^17^. Moroever, GluR2/3 subunits are found preferentially in neuronal exosomes^12^.

In this paper, we use intense synaptic activation in neuronal cultures to validate that our μMED device provides measurements similar to traditional exosome preparation techniques. We address the diagnostic value of profiling GluR2-containing exosomes using an *in vivo* mTBI model. We demonstrate the successful detection of brain-derived exosomes following mechanical injury to cortical neurons, after both cortical impact injury and blast-induced mTBI. Compared to conventional diagnosis of mTBI that is only based on patient reports and clinical symptoms, μMED offers a robust tool for monitoring of molecular-based changes in the brain over time after mTBI.

### GluR2 as a biomarker to monitor mTBI

One goal of this study is to demonstrate that a protein surface marker on isolated exosomes can be profiled to track the pathological state of the brain following mTBI. We selected the AMPA receptor subunit GluR2 that is expressed on the surface of exosomes, as this subunit is expressed widely in neurons of different types across the entire brain, as well as in developing oligodendrocytes. Evidence from animal models of subarachnoid hemorrhage, cortical impact brain injury, and ischemia has shown that GluR2 levels in brain tissue changes following injury, also suggesting this surface marker may capture the dynamic changes that occur in the brain after mTBI^15^,^18^. Furthermore, the overabundance of glutamate AMPA receptors has been implicated in the hyperexcitability, epileptic activity, calcium-dependent cell swelling, and cell death that occur in the secondary injury phase of mTBI^15^. Therefore, we hypothesized that monitoring the level of GluR2-containing exosomes that appear following mTBI could provide insight into the changes that appear both acutely and during the longer recovery phase after mTBI.

### Rapid microbead assay for exosome capture

The first step of the assay in our μMED device is to capture exosomes onto antibody functionalized microbeads, enriching for brain-derived exosomes that appear in blood serum. To minimize competition from abundant exosomes from healthy blood cells (leukocytes, platelets), background exosomes are first depleted using anti-CD45 and anti-CD61 coated 7 μm diameter microbeads (Fig. 1c). Remaining exosomes are captured using exosome-specific anti-CD81 coated 2.2 μm diameter microbeads (Fig. 1d). The exosomes are subsequently labeled with horseradish peroxidase (HRP) modified antibody that targets the AMPA receptor GluR2 (Fig. 1e). Subsequent to labeling, these affinity ligands are converted into a measurable fluorescence signal using enzyme amplification (Fig. 1f).

### μMED chip design

**Figure 2:**
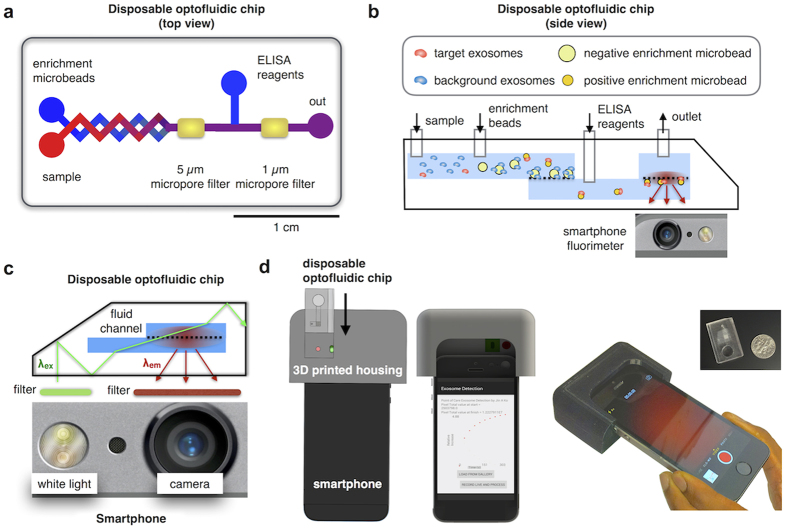
Design and Implementation of microfluidic-based Mobile Exosome Detector (μMED). (**a)** Top view of μMED, showing sample inputs, chaotic mixers where reagents are rapidly mixed and incubated on-chip, and the integrated micropore filters that are used to trap beads. (**b)** Side view of μMED, showing the step-by-step assay carried out on our chip. (**c)** Optical setup for μMED’s integrated fluorescence read out. Our design incorporates the bright LED and sensitive camera that are available on modern mobile phones. By combining these features with off-of-the-shelf optics, integrated into a custom 3D printed piece, we developed a mobile fluorimeter to read out the results of our exosome assay. (**d)** A CAD drawing of the integrated device, showing the disposable and the reusable piece as well as the smartphone and a photograph of the μMED in action.

On the μMED chip, this entire assay is miniaturized and automated, enabling practical clinical use and also conserving precious sample volume (Fig. 2a). The operation of the chip can be broken down into the following steps. First, negative selection beads (7 μm) and positive selection beads (2.2 μm) are mixed with serum and incubated for 30 minutes in an on-chip reservoir. The sample is subsequently pulled through the device using a single negative pressure supply (Braintree, Programmable Syringe Pump). The 7 μm negative selection beads are then captured onto an integrated 5 μm porous membrane, allowing only those exosomes that have not been captured onto the negative selection beads to pass. Downstream, the 2.2 μm positive selection beads are trapped onto a 1 μm porous membrane, where subsequent washing steps, labeling for injury-specific surface markers (*e.g.* GluR2), and enzyme amplification are performed (Fig. 2b). Following capture, the beads are washed with phosphate buffered saline (PBS) solution at a high flow rate (ɸ > 50 mL/hr) for ~10 seconds to wash the isolated microbeads. Additionally, the device is pre-treated with Pluronic F-127 to minimize non-specific binding of reagents or residues on chip that could cause background fluorescence. After washing the beads, the fluorescence substrate, QuantaRed Enhanced Chemifluorescent HRP substrate (Life Technologies), is introduced. At the time-point that the substrate is introduced, the fluorescence intensity recording begins on the smartphone based device. Background subtraction is performed by subtracting the fluorescence intensity at a time point immediately before the fluorescence substrate is introduced. All data analysis is carried out on a custom App installed in the smartphone. The entire chip is 1.9 [W] x 2.9 [L] x 0.8 [H] cm^3^, roughly the size of a US quarter. Because the device requires only one negative pressure supply and does not require accurate flow-control, it is well suited for mobile use where flow can be driven by vacuum pack or capillary action^19^,^20^,^21^.

The basic operating mechanism of the on-chip fluorescence detection is shown in Fig. 2c. μMED takes advantage of the bright light emitting diode (LED) and sensitive camera that are available on smartphones. By combining these features with off-of-the-shelf optics, integrated into a custom 3D printed piece, we have developed a mobile fluorimeter to read out the results of our exosome assay. A bandpass filter (λ_pass_ = 510–560 nm) is positioned over the smartphone’s LED in the integrated 3D printed piece (See Supplementary Information for a detailed 3D drawing). This filter is tuned to the excitation wavelength of the fluorophore λ_ex_ = 570 nm, which is coupled into the device using total internal reflection off-of the edge of the chip (Fig. 2c). The light within the microchip uniformly and intensely illuminates the fluid channel using anti-resonant coupling^22^,^23^.

The fluorescent dye generated by the enzyme amplified assay absorbs the excitation light and emits light (λ_em_ = 585 nm) isotropically into the smartphone camera where it is detected (Fig. 2c). Most of the excitation light is confined within the device due to total internal reflection. To further reduce the background of scattered excitation light, we position a dichroic filter over the smartphone camera. Our custom designed 3D printed piece integrates the smartphone and the microchip together, allowing the disposable microchip to be inserted and automatically aligned with the optical components and the smartphone for easy and reliable use. A smartphone App was written to control the excitation light source, measure the emitted light, and analyze the data, demonstrating our technology’s capability for use as a point-of-care medical diagnostic (Fig. 2d).

### Validation of exosome isolation using μMED

To validate that the μMED’s microbead-based assay isolated exosomes, we imaged the isolate from our assay using cryological transmission electron microscopy (cryo-TEM) (University of Pennsylvania School of Medicine, Electron Microscopy Resource Laboratory). In these imaging experiments, we used our microbead assay to isolate exosomes from our *in vitro* mTBI murine cell culture model^41^. Next, we used cryo-TEM to image exosomes on the surface of the CD81 functionalized microbeads (Fig. S3a). We confirmed that bound to these 2.2 μm diameter beads were ~100 nm diameter objects with morphology consistent with that of exosomes. Additionally, we performed cryo-TEM imaging on exosomes eluted from those microbeads onto a TEM grid. In these images (Fig. S3b), we were also able to see ~100 nm vesicles with morphology consistent with exosomes.

We further validated that the μMED isolated exosomes by measuring both the chip’s input and its isolate using Nanoparticle Tracking Analysis (NTA) (Nanosight, Malvern; Nanomedicine Characterization Core Facility, University of North Carolina). We isolated exosomes from our *in vitro* mTBI murine cell culture model^41^ using our microbead assay. The input, unprocessed cell culture media, (Fig. S3c) was found to contain a concentration of 2.88 × 10^10^ exosomes/mL with a mean diameter <d> = 113.8 nm and a standard deviation σ = 31.3 nm. The isolate, exosomes captured by our microbead system and then eluted for NTA measurement, (Fig. S3d) was found to contain a concentration of 3.34 × 10^10^ exosomes/mL, exosomes with a mean diameter of <*d*> = 116.6 nm, and a standard deviation of σ = 37.3 nm. The input and output have size distributions and concentrations consistent with those of exosomes^11^.

### An Increase of the GluR2+ exosome level following mechanical injury to cortical neurons

**Figure 3:**
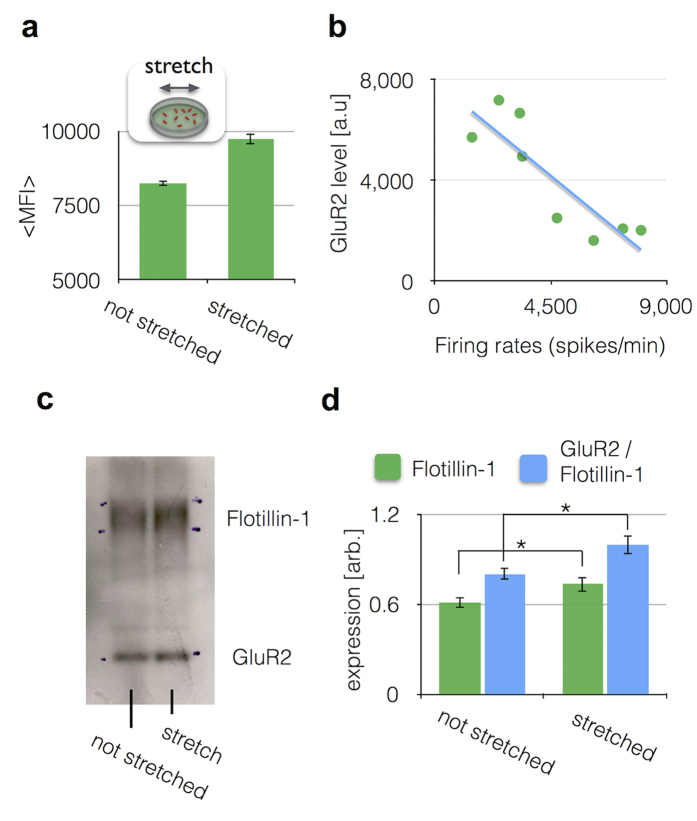
Validation of μMED assay using an *in vitro* model for mTBI. (**a**) Using an *In vitro* stretch model of TBI, N = 9 stretched and N = 11 not stretched experiments were performed. Error bars signify standard error. (**b**) We quantitatively compared the GluR2 level in isolated exosomes with the Calcium imaging activity level. (**d**) The line average of Flotillin-1, representing the total amount of exosomes, and GluR2/Flotillin-1, representing the amount of GluR2 per exosome (*P < 0.05, unpaired Student’s T test). Error bars represent Standard Error of the Mean (SEM).

Past work shows that cultured neurons will show a significant reduction in activity following a single, brief mechanical injury^40^ that mimics the mechanical forces that occur within the brain during mTBI^43^. Using our *in vitro* mTBI murine cell culture model^41^, we first correlated exosomal biomarker release with estimates of neuronal activity that occurred within the first ninety minutes after injury. We used a cell stretch model of injury that does not cause immediate or delayed (24 hr) neuronal death, modeling the relative absence of cell death that occurs following mTBI. Ninety minutes after a single injury, we measured neuronal activity with calcium imaging and compared it to the GluR2 exosome level measured by μMED from the cell culture media. A 20% increase in GluR2+ exosomes was detected in samples from wells that experienced a stretch injury *versus* those that did not.(p = 0.003) (Fig. 3a). We validated the specificity of our antibody-based capture (CD81) and our antibody-based labeling (GluR2) by performing control measurements on cultured media from our cortical neuron *in vitro* model, wherein we iteratively replaced each antibody in our assay with a control antibody (Fig. S2a).

To demonstrate that GluR2+ exosomes could be used to monitor neuronal activity following mTBI, we measured neuronal activity (spikes/min) before and after injury using calcium imaging and compared it to the quantity of GluR2+ exosomes measured. We averaged activity measurements over one minute, inspecting 50–150 neurons per field of view. We quantitatively compared the GluR2 level in isolated exosomes with the calcium imaging activity level after injury and found a negative correlation (R^2^ = 0.748) (Fig. 3b). As exosomal GluR2 level increased, the neuronal activity started to decrease.

We validated μMED by comparing its results to those measured with conventional methods. To this end, we compared the performance of μMED to conventional size-based isolation (centrifugation) and Western Blot. We isolated exosomes from cell culture media, from both stretched and not stretched samples, using ultracentrifugation and subsequently measured using Western blot. In our Western blot measurement, in addition to GluR2, we also measured Flotillin-1, which we used as a measure of total exosome count^26^ (Fig. 3c). To quantitatively compare the expression of Flotillin-1 and GluR2/Flotillin-1, we calculated line averages of the Western Blot data in Fig. 3b using Image J (Fig. 3d). We found that cultured cortical neurons released significantly more exosomes (Flotillin-1) (*p* = 0.04), and more GluR2 per exosome (GluR2/Flotillin-1) (p = 0.02) in response to a stretch injury that mimics mTBI (Fig. 3f). These results were consistent with the increase in GluR2+ exosomes measured by μMED after stretch injury (Fig. 3c).

### Combined negative and positive microbead isolation of exosomes directly from serum

To isolate exosomes directly from unprocessed serum, we used functionalized microbeads (Fig. 1c,d) which confers several key advantages: 1. Exosomes can be selectively and rapidly isolated (<1 hr) without the use of lengthy (24 hrs) and bulky laboratory equipment (i.e. ultracentrifuge), thus making exosome diagnostics practical for routine clinical use. 2. The use of affinity ligand functionalized microbeads enables exosomes to be isolated with reduced copurification of non-exosomal debris and exosomes from abundant healthy blood cells (e.g. leukocytes, platelets), compared to size-based isolation schemes. 3. Bead-based isolation enables negative selection of exosomes from abundant cell types (leukocytes, platelets) prior to positive selection to diminish background, which would otherwise limit sensitivity.

By using negative selection, the μMED assay was able to selectively profile brain derived exosomes directly from unprocessed serum. To minimize competition from abundant exosomes from healthy blood cells (leukocytes, platelets) in our assay, background exosomes are depleted *via* a negative selection step using anti-CD45 and anti-CD61 coated 7 μm diameter microbeads (Fig. S5). One potential problem with this assay is that the positive selection beads, which are functionalized with a pan-exosome marker, will also capture exosomes that are in the background. However, we found that there is not a significant quantity of background exosomes captured by the positive selection beads (Fig. S5). We postulated that this is because there are >500 x more negative selection beads than positive selection beads, and thus the majority of the background exosomes are captured on the negative selection beads. Additionally, due to the final step in our assay, wherein we label the exosomes captured by the positive selection beads with GluR2, the non-brain derived exosomes do not contribute to our output signal. Moreover, rather than performing positive and negative selection simultaneously, the negative selection step and positive selection step can also be carried out sequentially, which eliminates this concern.

**Figure 4:**
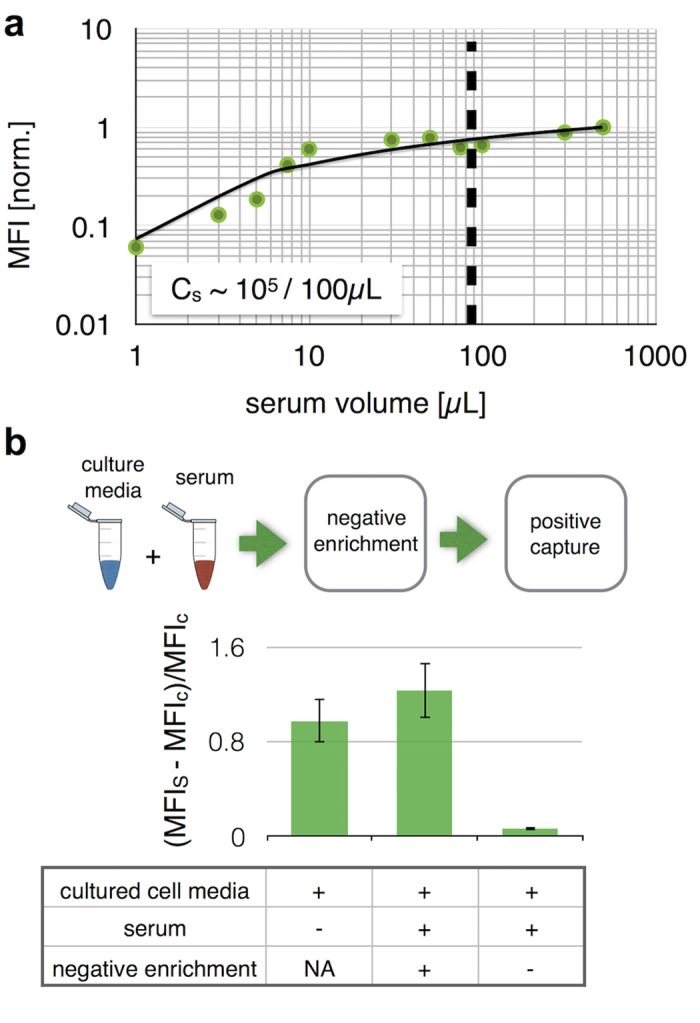
Combined negative and positive microbead-based exosome capture. (**a**) anti-CD45 and anti-CD61 beads were used to deplete background exosomes present in serum. Bead number was held constant (*N* = 10^5^) and a varying volume of serum was spiked into buffer, demonstrating that C_s_ = 10^5^ beads/100 μL was required to adequately clear the vast background of CD45 and CD61 expressing exosomes. (**b**) By combining negative and positive selection, brain derived exosomes could be profiled directly from unprocessed serum. The μMED assay was performed on cortical neuron cultured media spiked into healthy mouse serum, as well as a positive control wherein the complete μMED assay is performed on cortical neuron cultured media without serum. And, a negative control is performed in which negative selection is removed from the μMED assay. The result is reported as the fractional change of mean fluorescence intensities (MFI_s_ -MFI_c_)/MFI_c_ where MFI_s_ and MFI_c_ are the mean fluorescence intensity from the sample and a negative control (uncultured fresh media) respectively. Error bars represent Standard Error of the Mean (SEM).

We demonstrated that negative and positive enrichment can be combined to capture targeted exosomes directly from serum. To this end, we first demonstrated that anti-CD45 and anti-CD61 beads can be used to deplete background exosomes present in serum (Fig. 4a). By using a constant number of anti-CD45 beads (*N* = 10^5^) and a varying volume of serum, we found that C_s_ = 10^5^ beads/100 μL was required to adequately clear the vast background of CD45 and CD61 expressing exosomes. This calibration data allowed us to choose the number of beads required for a given volume of serum sample.

To test the efficacy of this negative selection step, we performed the μMED assay on exosomes harvested from cortical neuron cultured media and spiked into healthy mouse serum (Fig. 4b). As a negative control, we performed the same assay with the negative selection step removed. And, as a positive control we performed the complete μMED assay directly on the cortical neuron cultured media. We quantified the results of these measurements by reporting the fractional change in the quantity of the fluorescence signal measured compared to the signal that arose when the cortical neuron cultured media was replaced with fresh media. The signal is reported as the fractional change of mean fluorescence intensities (MFI_s_ -MFI_c_)/MFI_c_ where MFI_s_ and MFI_c_ are the mean fluorescence intensity from the sample and a negative control (uncultured fresh media) respectively. By using negative selection, the μMED assay was able to detect brain derived exosomes directly from unprocessed serum, obtaining results that matched those obtained directly on cortical neuron cultured media.

### Device operation

**Figure 5:**
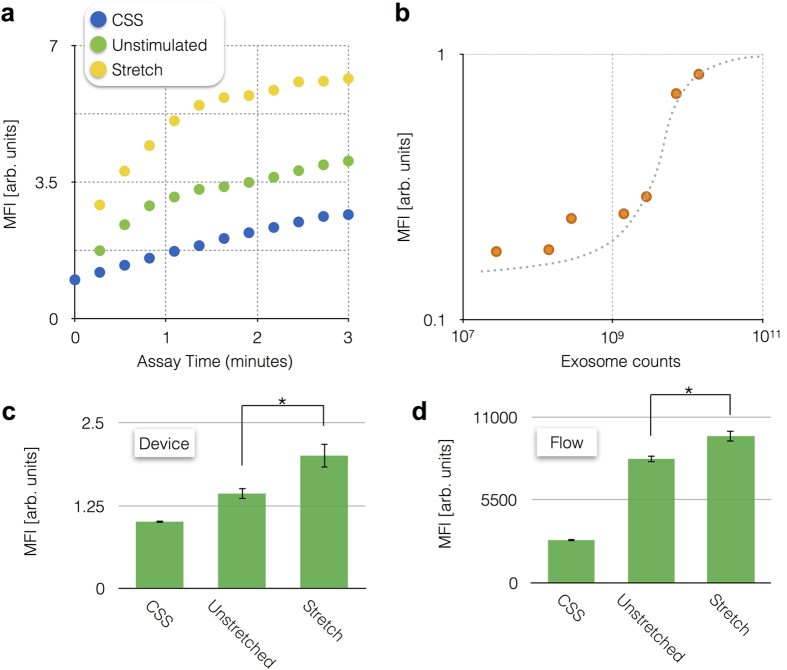
μMED Device operation. (**a**) μMED was validated by performing the enzyme amplification assay on-chip and comparing the results to flow cytometry on the assay’s microbeads. (**b**) We performed a serial dillution of cultured media from our cortical neuron *in vitro* model to measure the limit of detection. Device response was compared for N = 4 stretched, N = 4 not stretched, and N = 4 negative controls on both the μMED (**c**) device and on flow (N = 9 stretched, N = 11 not stretched, and N = 7 negative controls) (**d**), which showed qualitatively similar results. Error bars represent Standard Error of the Mean (SEM). *P < 0.05.

μMED’s ELISA fluorescence detection was validated by performing the enzyme amplification assay on-chip and comparing the results to flow cytometry on the assay’s microbeads. On the chip, the enzyme amplification was tracked over a 3 min time period and it was found that stretched samples lead to greater increasing rate (Fig. 5a) and amplitude (Fig. 5c) of the enzyme-amplified fluorescence signal. The results were consistent with those obtained by measuring the same samples using flow cytometry (Fig. 5d). Device response was compared for N = 4 stretched, N = 4 not stretched, and N = 4 negative controls on both the μMED device and on flow (N = 9 stretched, N = 11 not stretched, and N = 7 negative controls), which showed consistent results. As a baseline control, we used control salt solution (CSS), which is fresh media without exosomes. For both flow cytometry and the μMED, the CSS sample led to a significantly lower signal than either the stretched or unstretched sample (P < 0.05). Because μMED measures the increase in fluorescence signal *versus* time, the duration of time that the beads are exposed to the fluorescent substrate did not have to be optimized.

To experimentally determine the limit of detection (LOD) of the μMED, we measured a set of samples with decreasing concentrations of exosomes. We prepared these samples by first independently measuring the concentration of exosomes in a sample of cultured media from our cortical neuron *in vitro* model using nanoparticle tracking analysis (NTA). Using these samples, we created a set of serially diluted samples ranging from 10^7^ to 10^11^ exosomes/mL, which we measured using the μMED (Fig. 5b). The LOD was found to be 10^7^ exosomes, which is more sensitive than commercial exosome ELISA kits and similar to comparable microfluidic platforms^11^.

### Increased level of GluR2+ exosomes from Controlled Cortical Impact (CCI) and Blast injured mice

**Figure 6:**
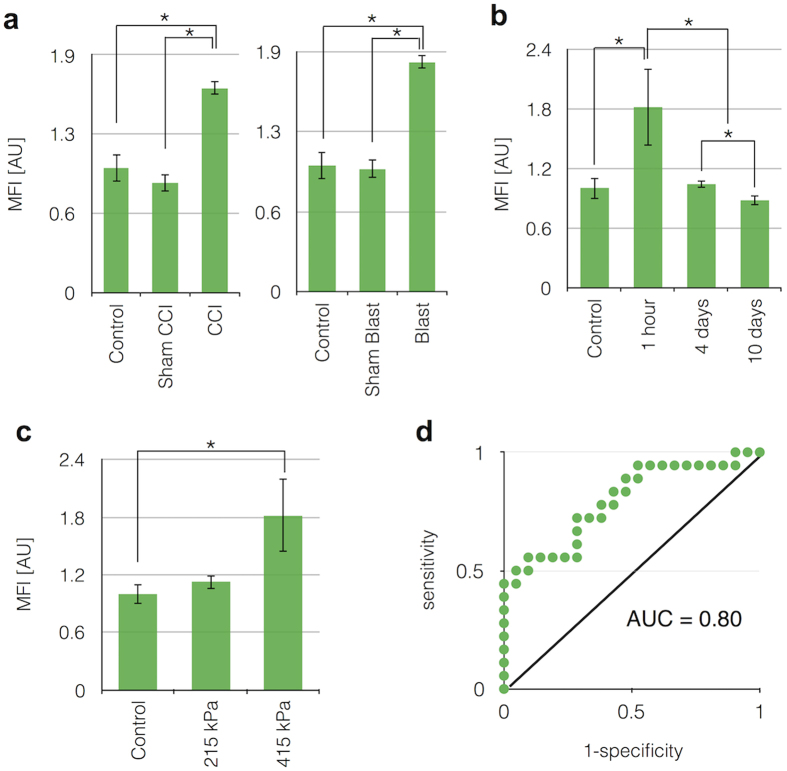
*In-Vivo* testing of assay using controlled cortical impact and blast murine models. (**a**). Using a mouse model for CCI and blast injury, we demonstrated that our assay could detect an increased level of GluR2+ exosomes following mTBI in N = 8 CCI and N = 9 blast mice, compared to N = 4 sham CCI and N = 5 blast injury models, as well as N = 9 naïve control mice. (**b**). The transient response of exosome biomarkers to an mTBI was measured at 1 hour (N = 12), 4 days (N = 4), and 10 days (N = 3) after injury. The GluR2+ exosome level dropped over the course of 4 days, and reached its baseline level by day 10. (**c**). Additionally, the GluR2+ exosome level was measured for varying levels of blast exposure. As expected, moving from a weaker (215 kPa peak pressure) (N = 4) to a stronger (415 kPa peak pressure) (N = 12) blast, there was a significant increase in GluR2+ exosomes. (**d**). To characterize the tradeoff between sensitivity and specificity, we tested the device using a range of threshold values Ψ_t_, above which we identify a mouse as having had an mTBI. We vary this threshold to generate a Receiver Operator Characteristic (ROC) curve, with an AUC = 0.80. *P < 0.05 (one way ANOVA test followed by a post hoc pairwise comparison using HolmSidak method (alpha = 0.05)).

Using two different models of mTBI – controlled cortical impact (CCI)^27^ and exposure to a controlled shock wave (blast)^28^ – we demonstrated that our assay could detect an increased level of GluR2+ exosomes following mTBI (Fig. 6a). We compared serum samples from brain injured mice to sham CCI and sham blast animals, where the surgery to prepare the mice was performed but the injury was not. Additionally, we measured naïve control mice that received neither injury nor the sham surgery preparation. We observed an average of 1.8x fold increase on the GluR2+ exosome level due to mTBI (including both CCI and blast) compared to control (p = 0.01), validating that our diagnostic could resolve exosome biomarkers for mTBI in an *in vivo* system. To validate the specificity of our antibody-based capture (CD81) and our antibody-based labeling (GluR2) in these experiments, we performed control measurements on murine serum samples, wherein we iteratively replaced each antibody in our assay with a control antibody (Fig. S2b). To further validate our results, we compared our results to conventional methods, wherein we isolated exosomes using a conventional size-based method (Total Exosome Isolation Kit, Life Technologies) and profiled the exosome content using Western Blotting. Consistent with the results of the μMED, GluR2/Flotillin1 expression of the isolate was significantly increased in the mice that had experienced injury (*N* = 6 pooled samples) relative to the healthy control (*N* = 6 pooled samples) (Fig. S4), though less pronounced than what was measured on our device. However, we could not detect a change in overall exosome level, as measured by profiling Flotillin-1. This result is consistent with our expectations, as size-based sorting results in an increase in copurification and did not have the benefit of μMED’s negative selection step to enrich for brain-derived exosomes.

The transient response of exosome biomarkers to an mTBI was measured at 1 hour, 4 days, and 10 days after injury (Fig. 6b). The GluR2+ exosome level dropped over the course of 4 days, and reached its baseline level by day 10. The GluR2+ exosome level for 4 days after injury was not significantly different (p = 0.28) from 1 hour after injury. However, 10 days after injury showed significant difference of GluR2+ exosome level compared to 4 days after injury (p = 0.03). Additionally, the GluR2+ exosome level was measured for varying levels of blast exposure (Fig. 6c). Moving from a weaker (215 kPa peak pressure; 46 kPa^*^msec impulse) to a stronger injury (415 kPa peak pressure; 148 kPa*msec impulse) caused an increase in GluR2+ exosomes.

We evaluated the sensitivity and specificity of signal measurements of injured mice relative to uninjured controls. We tested a variety of threshold values Ψ_t_, above which we would label a mouse as having had an mTBI and below which we would label the mouse as being uninjured. To characterize the tradeoff between sensitivity and specificity, we tested the device using a range of threshold values Ψ_t_ and generated a receiver operator characteristic (ROC) curve (Fig. 6d). We defined the sensitivity = TP/P, where TP is the number of instances the chip successfully correctly identified a mouse that had mTBI. We defined the specificity = TN/N, where TN = N − FP is the true negatives and is defined by the total false positives FP and the total number of uninjured mice N. We found an area under the curve, AUC = 0.80, demonstrating the ability to detect mTBI based only on a blood test, allowing for inherent variability of individual mouse. For the threshold value Ψ_t_ chosen, the sensitivity was 73% and the specificity was 71%. In order to avoid operator-bias, the sample collection and diagnostic test data collection were done in double-blinded fashion.

## Discussion

Our smartphone-based mTBI diagnostics, μMED, offers rapid, portable, and cost effective technology that can monitor mTBI and prognose secondary brain injury, which are otherwise extremely expensive (brain MRI ~$5000) and hard to predict. The combination of smartphone-based and serum-based detection on our microchip provides a new mTBI diagnostic that is suitable for clinical settings.

Existing platforms that use microfluidics to selectively and sensitively sort and detect cells face technical challenges when sorting and measuring microvesicles and exosomes, due to the expense of nanolithography, the inherently low throughput and susceptibility to clogging of nanoscale fluid channels, and the unfavorably strong scaling of many of the forces used to sort objects in microfluidics as they become nanoscale. In recent years, new creative approaches emerged to harness micro/nano-devices to isolate and detect exosomes, including those that sort exosomes based on their size^11^,^29^ or based on their surface markers^30^,^31^,^32^,^33^. In contrast to strategies that isolate exosomes based on their size, immunoaffinity-based isolation used in this work reduces copurification with cell-debris and protein aggregates as well as enables the ability to isolate specific subpopulations of exosomes or microvesicles based on the expression of a specific surface marker^30^,^31^,^32^,^33^. Our work is most differentiated from previous approaches, based on in its achievement of using only smartphone-based optics to read out exosome biomarkers, enabling point-of-care detection directly from serum samples.

The point-of-care μMED chip enables rapid isolation and detection of brain-derived exosomes from serum of mTBI mice. We demonstrated that the technology offers versatility for detecting changes following different types of injury that includes contusive brain injury (CCI) and concussive brain injury (blast TBI). A natural extension of this technology is profiling the contents of the exosomes for cytoskeletal proteins currently under evaluation for use as TBI biomarkers (e.g., Tau, neurofilament, spectrin breakdown products)^34^. Of course, the variety of molecular species contained within exosomes also offers an opportunity to explore new categories of TBI biomarkers that include both mRNA and microRNA changes, as well as alterations in lipid content, given the past work showing that each of these factors can change after TBI^35^,^36^,^37^,^38^. The portability and affordability of the platform also raise the possibility of longitudinal clinical studies combining exosomal biomarkers with advanced brain imaging during recovery of mTBI to measure the molecular dynamics of cognitive recovery in more detail. Extracting exosomes that originate in a specific target organ from a blood sample also presents an exciting diagnostic potential for additional diseases beyond TBI. The specificity of these tests will rely heavily on the identification of exosome surface markers that are unique to the relevant tissue or cell type. Additionally, due to the modular nature of our chip, it can be further improved to profile multiple exosomal biomarkers at once on the same chip resulting in an increasingly specific and detailed picture of the recovering brain^7^. These features allow the μMED chip to be a powerful tool suitable for practical use in the clinic.

## Methods

### Cell culture

To test our device, we used an *in vitro* cortical neuron model. Embryos at day E18 were surgically removed from a timed pregnant Sprague-Dawley rat anesthetized with 5% CO_2_ and sacrificed via cervical dislocation. Neocortical tissue was dissected from the embryos and dissociated for 15 min at 37 °C in trypsin (1.4 mg/mL) and DNAse (0.6 mg/mL, Roche Applied Science, Indianapolis, IN). After trituration and filtration through Nitex mesh (Crosswire Cloth, Bellmawr, NJ), cells were resuspended in MEM with Earle’s salts and GlutaMAX supplemented with 0.6% d-glucose (Sigma–Aldrich, St. Louis, MO), 1% Pen-Strep, and 10% Horse Serum and plated on poly-d-lysine- (0.08 mg/mL, Sigma–Aldrich) and laminin- (0.001 mg/mL, BD Biosciences, San Jose, CA) coated silicone deformable membranes (.005″ Gloss/Gloss, Specialty Manufacturing Inc, Saginaw, Mi). Cells were plated at a density of 400,000 cells/mL, roughly 20,000 cells/mm^2^. After overnight adhesion, media was replaced with Neurobasal media supplemented with B-27 and 0.4 mM GlutaMAX and grown in a humidified 37 °C, 5% CO_2_ incubator. The University of Pennsylvania office of University Laboratory Animal Resources (ULAR) oversees all work with rats. All experimental protocols were approved and animal care and use was in accordance with the guidelines specified by the Institutional Animal Care and Use Committee (IACUC) of the University of Pennsylvania.

### Calcium imaging and data acquisition

Neurons were transduced with a genetically engineered calcium indicator (GECI) at least 7 days prior to planned imaging study with an adeno-associated virus expressing GCaMP6f under the control of the synapsin-1 promoter^39^ (Penn Vector Core #AV-1-PV2822, GC 5.92*10^9^). Time-lapse calcium imaging of a neuronal population (50–150 neurons in field of view; 20 Hz framing rate) was measured preinjury and 60–90 min postinjury with a Nikon Eclipse TE2000U microscope fitted with a spinning disk confocal (CSU-10b, Solamere Technologies), a CCD camera (Photometric Cool-Snap HQ2, BioVision, Exton PA), 488-nm excitation laser (Prairie SFC), and a Nikon 10X Plan Apo objective (N.A. = 0.4). Exposure time was set to 50 ms, and images were streamed at 20 Hz frame rate for 1 min. Each image frame was 520 × 696 pixels, which corresponded to 0.3 mm^2^ rectangular area. Image processing and data analysis were performed with custom MATLAB scripts per our recent report^40^.

### Stretch injury

Cells were subjected to a strain-controlled stretch injury as described previously^41^. Briefly, cells plated on deformable membranes were inserted into a closed pressure chamber. A controlled air pressure pulse was injected into the chamber, causing the membrane to deflect through a rectangular opening (2.5 ∗ 18 mm^2^), and mechanically injure the cells only within this rectangular region. The magnitude of the stretch injury was calibrated to the applied input pressure for each week of experiments, and a transducer recorded the applied output pressure of each test (Endevco, San Juan Capistrano, CA). This resulting output pressure can be correlated with similar magnitude and strain rates observed in animal models of TBI^27^,^28^.

### Off chip micro-bead assay and flow cytometry

To independently validate and optimize our assay before integrating it onto the chip, we first implemented it using conventional laboratory equipment. Plasma samples were first incubated with anti-CD45 and anti-CD61 functionalized beads (BioLegend, Spherotech, VFP-2052-5) for 30 mins at room temperature (RT). The avidin-biotin system was used to allow a strong binding of avidin microbeads to biotinylated antibodies. For off-chip testing, the sample was then centrifuged at 3000 g for 10 mins and the supernatant was incubated with anti-CD81 functionalized beads (BioLegend, Spherotech, VP-60-5) for 30 mins at room. The sample was washed and incubated with anti-GluR2 antibody (Bioss) for 30 mins at RT. Then, the sample was incubated with HRP conjugate IgG antibody (Life Technologies) and with QuantaRed Enhanced Chemifluorescent HRP substrate (Life Technologies) right before running through LSR II Flow Cytometer (BD Biosciences). After data collection, FlowJo software was used for data analysis.

### Device fabrication

The microfluidic chip was made of Polydimethylsiloxane (PDMS), and was fabricated using a combination of casting using a 3D printed mold and laser micro-machining. To fabricate this device, first a mold was created for it using the Dimension Elite 3D printer (CAD drawing included in Supplementary Information). After silanization of the mold using Trichloro (1H,1H,2H,2H-perfluorooctyl) silane in a desiccator for 30 mins, to reduce adherence of the surface to PDMS, PDMS was poured into the mold and cured at 65 °C for 2 hours. After curing, microfluidic channels were engraved using laser micromachining (Universal Laser VLS 3.50). The molded PDMS piece, the polycarbonate track-etched filters (Whatman, Nucleopore), and a millimeter thick PDMS base were integrated together using stamping and bonding with uncured PDMS^44^,^45^. To test the device, we connected it to a syringe pump using Tygon microbore tubing (500 μm ID ×1.5 mm OD) inserted into a hole made by a biopsy punch (Miltex). The inputs (i.e sample and reagents) were loaded into a reservoir made using a biopsy punch. For mobile use, reagents can be pre-loaded onto the device^46^. The device was pre-treated with Pluronic F-127 (Sigma-Aldrich) to minimize non-specific retention of exosomes to the channel walls or to the track-etched filter.

The 3D printed piece, which incorporates optics, couples our device to the smartphone and aligns the disposable microfluidic chip with the optics, is created using a SolidWorks design and the Dimension Elite 3D printer available in the University of Pennsylvania’s Additive Manufacturing Lab, using the Dimension Elite machine. A bandpass filter (Omega Optical, 535QM50; λ_pass_ = 510–560 nm) was aligned on top of the smartphone LED and a red dichroic filter (Edmund Optics, λ_cut-on_ = 605 nm) was aligned with the smartphone camera.

### Ultracentrifugation

Total Exosome Isolation Kit (Life Technologies) was used to isolation exosomes using ultracentrifugation. Cell cultured media was mixed with the Total Exosome Isolation reagent and incubated at 4 °C overnight. After incubation, the mixed sample was centrifuged at 10,000 g for 1 hour at 4 °C. Then, the supernatant was discarded and the pellet was resuspended in 1X PBS. The exosome pellet was stored at 4 °C until usage.

### Western blotting

The exosomes were isolated from cultured medium utilizing a Total Exosome Isolation Reagent Kit (ThermoFisher Scientific, cat. #4478359). The isolated exosome samples were divided equally into labeled tubes. The samples were spun down into a pellet in Beckman Coulter Optima L-100K Ultracentrifuge with a SW 60 Ti hanging bucket rotor at 4 °C at 50,000 g for 3 hours. The pellet was resuspended in a minimal amount (25 –50 μl) of RIPA Buffer with HALT Protease and Phosphatase Inhibitor Cocktail added (Thermo Scientific, cat. #78440). An aliquot of the resuspended exosome samples was mixed with an appropriate amount of NuPAGE LDS 4X Sample Buffer (Life Technologies/Thermo Fisher Scientific, cat. #NP0007). 30 μl of the prepared samples was run on a NuPAGE 10 well 4–12% BIS-TRIS Mini Gradient Gel (Life Technologies/Thermo Fisher Scientific, cat. #NP0335) with 1X MES Running Buffer (Life Technologies/Thermo Fisher Scientific) at 125 constant Volts for 1 hour. The gels were then transferred onto preconditioned 0.2 Nitrocellulose Membrane (Life Tecnologies/Thermo Fisher Scientific, cat. #LC2000), in an XCell II™ Blot Module at 24 constant Volts for 1 hour. The Nitrocellulose blot was then blocked overnight at 4 °C, on a rocker with a 5% BSA TBS/0.1% Tween solution. Next, the blot was then probed overnight, on a rocker at 4 °C, with two primary antibodies; anti-GluR2/3 (EMD Millipore, cat. #07-598) and anti-Flotillin-1 (BD Transduction Laboratories, cat. #610821), diluted in blocking buffer at a concentration of 2 μg/ml and 1:1000 respectively. Finally, the blot was then probed with an HRP-linked Clean Blot Secondary Antibody (Thermo Fisher Scientific, cat. #21233) at 1:400 diluted in blocking solution 5% BSA TBS-T (0.1% Tween). To visualize the band, the blot was first incubated with Pierce ECL PLUS Western Blotting Detection Substrate (Thermo Fisher Scientific, cat. #32132). Amersham Hyperfilm ECL (GE Healthcare LTD, cat. #28906838) was exposed to the chemiluminescent blot. The exposed film was next developed in Kodak GBX Developer (Sigma-Aldrich, cat. #1900984) and fixed in CareStream GBX Fixer (Sigma-Aldrich, cat. #1902485). Finally, the dry exposed film was analyzed using ImageJ to measure the density of the bands of interest; Flotillin-1 at ~45 kDa and GluR2/3 at ~110 kDa.

### Mouse injury - CCI and blast

Controlled cortical impact and blast exposure experiments were performed on adult male (12–14 week old) C57BL/6J mice (Charles River, MA). All CCI injuries were performed as previously described (refs 27 and 47). Briefly, mice were anesthetized with isoflurane (3.0% induction, 1.5–2.0% maintenance in medical grade air: 21% oxygen, 78% nitrogen) and placed in a stereotaxic frame. A manual craniotomy was performed on the right parietotemporal region of the skull midway between bregma and lambda using a 4 mm diameter trephine (CMA7431058, Harvard Apparatus, Holliston, MA). The skull flap was removed and the animal was loaded into the CCI machine. Isoflurane was stopped 30 sec before CCI. A moderate injury was produced at an average impact velocity of 2.4 m/sec with an impact depth of 1.0 mm, centering the impact at approximately −2.5 mm bregma. Sham control animals underwent craniotomy, placed in the stereotaxic holder, but no CCI was delivered. Following the CCI, the exposed area was sutured and mice were allowed to emerge from anesthesia in a heated cage.

All Blast injuries were performed as previously described (ref. 28, PMID: 27246999). Briefly, mice were anesthetized with isoflurane (3.0% induction, 1.5–2.0% maintenance in medical grade air: 21% oxygen, 78% nitrogen). Sound deadening foam was inserted into each ear for protection. The mice were loaded into a holder positioned 1 cm away from the end of the shock tube oriented such that the blast wave travelled along the rostral-caudal axis of the head, i.e., with their snouts facing the shock tube. Mice were enclosed in a plastic housing and head motion was constrained with a thin metal rod encircling the snout and a cervical collar positioned between the occiput and shoulders. Isoflurane was stopped 30 sec before blast. A single pressure pulse was administered. High level injuries corresponded to a peak overpressure of 415 +/−41 kPa for 1.02 +/−0.04 ms whereas low level injuries corresponded to 215 +/−13 kPa for 0.65 +/−0.04 ms. After blast, animals were placed supine and monitored until they rolled onto their stomach. This time was recorded as the righting time. Sham animals underwent all aspects except for exposure to the pressure pulse. The University of Pennsylvania office of University Laboratory Animal Resources (ULAR) oversees all mouse work. All experimental protocols were approved and animal care and use was in accordance with the guidelines specified by the Institutional Animal Care and Use Committee (IACUC) of the University of Pennsylvania.

## Additional Information

**How to cite this article**: Ko, J. *et al.* Smartphone-enabled optofluidic exosome diagnostic for concussion recovery. *Sci. Rep.* **6**, 31215; doi: 10.1038/srep31215 (2016).

## Supplementary Material

Supplementary Information
