# Supplementary material for: Smartphone-enabled optofluidic exosome diagnostic for concussion recovery
Source: Sci Rep. 2016 Aug 8;6:31215. doi: 10.1038/srep31215 (PMC4976377; doi:10.1038/srep31215)
Supplement: Supplementary Information [file srep31215-s1.pdf]

Smartphone-enabled optofluidic exosome diagnostic for concussion recovery

Jina Ko[1], Matthew A. Hemphill[1], David Gabrieli[1], Leon Wu[1], Venkata Yelleswarapu[1], Gladys Lawerence[1], Wesley Pennycooke[1], Anup Singh[1], Dave Meaney[1*], David Issadore[1,2*]

1. Department of Bioengineering, School of Engineering and Applied Sciences, University of Pennsylvania. Philadelphia, Pennsylvania, United States.
2. Department of Electrical and Systems Engineering, School of Engineering and Applied Sciences, University of Pennsylvania. Philadelphia, Pennsylvania, United States.

* equal contribution

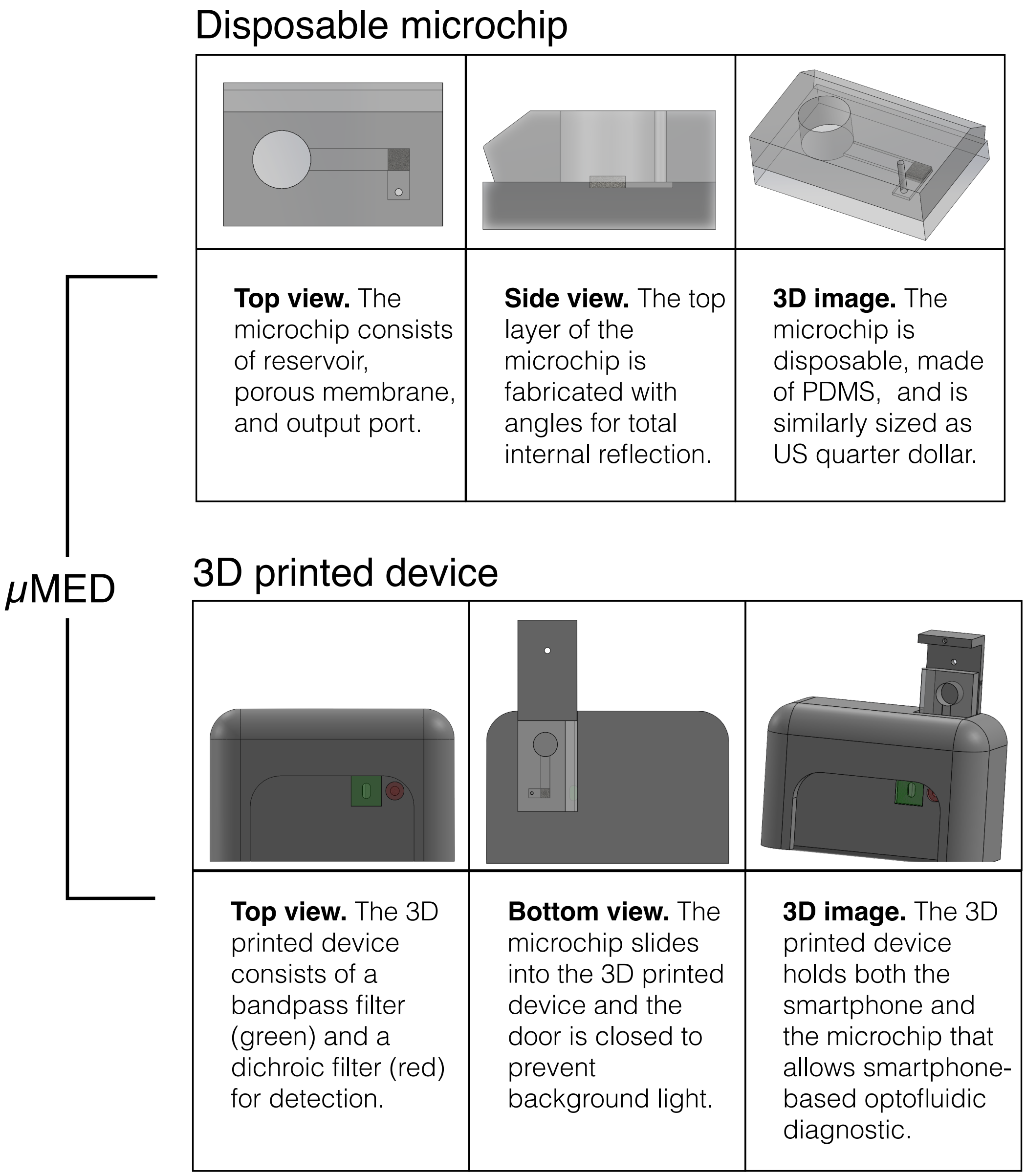

Figure S1. Computer Assisted Design (CAD) drawings of our *μ*MED device.

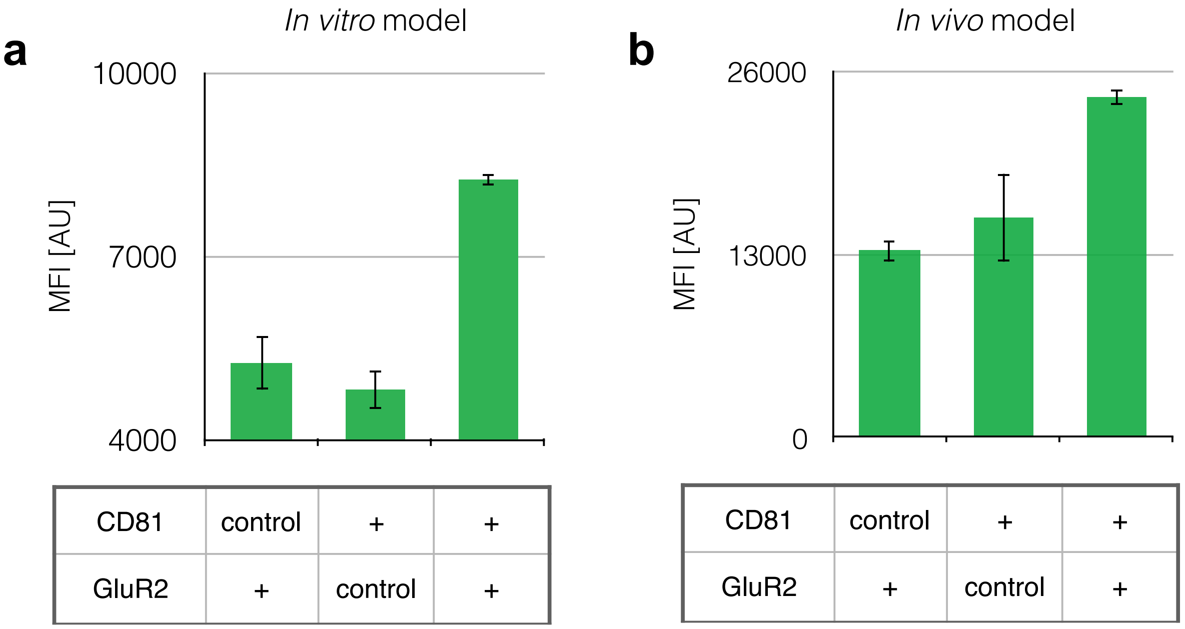

Figure S2. Validation of antibody specific capture and labeling. a. We validated the specificity of our antibody based capture and labeling using V = 1 mL of cultured media from our cortical neuron *in vitro* model. To test if the CD81 microbead capture of exosomes is specific, we replaced the CD81 pan exosome antibody with a control antibody (purified rabbit polyclonal Isotype control antibody) and saw a significant reduction in μMED's signal (MFI). To test if the labeling of our exosomes with GluR2 is specific, we replaced the GluR2 antibody with a control antibody (purified rabbit polyclonal Isotype control antibody) and saw a significant reduction in signal in μMED's signal (MFI). **b.** We performed the same experiment, and saw similar results validating the specificity of our capture and labeling, using 100 μL of serum from our *in-vivo* murine model.

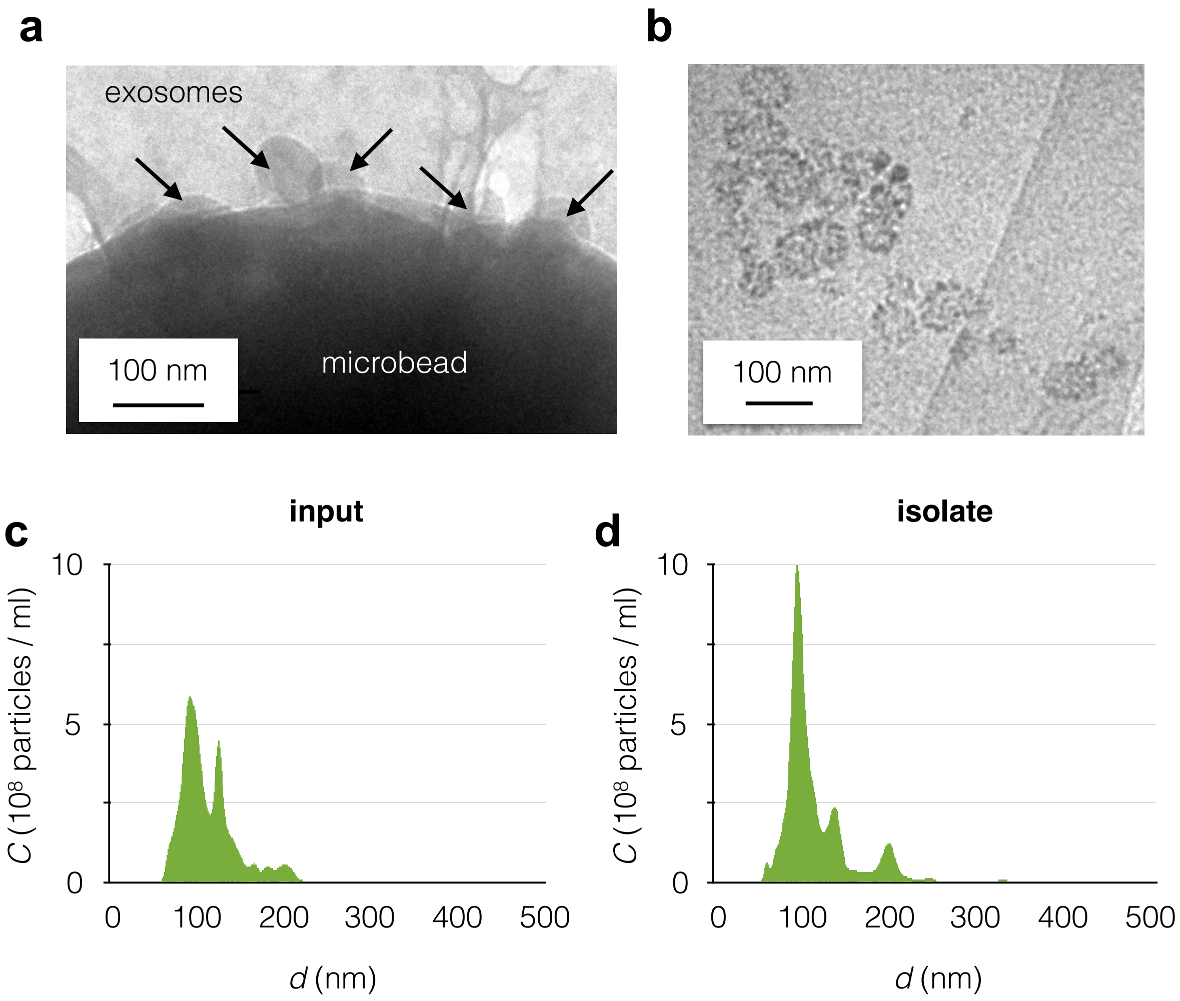

Figure S3. Characterization of isolated exosomes. a. A cryological Transmission Electron Microscope (cryo-TEM) micrograph of individual exosomes bound to the CD-81 functionalized 2.2 μm polystyrene bead used to capture it. The exosomes were captured from cultured cortical neuron cultured media. **b.** cryo-TEM imaging on exosomes eluted from those microbeads onto a TEM grid. **c.**Nanoparticle Tracking Analysis (NTA) data of exosomes from unprocessed cortical cell culture media. **d.** NTA data of the exosomes captured by our microbead system and then eluted for NTA measurement.

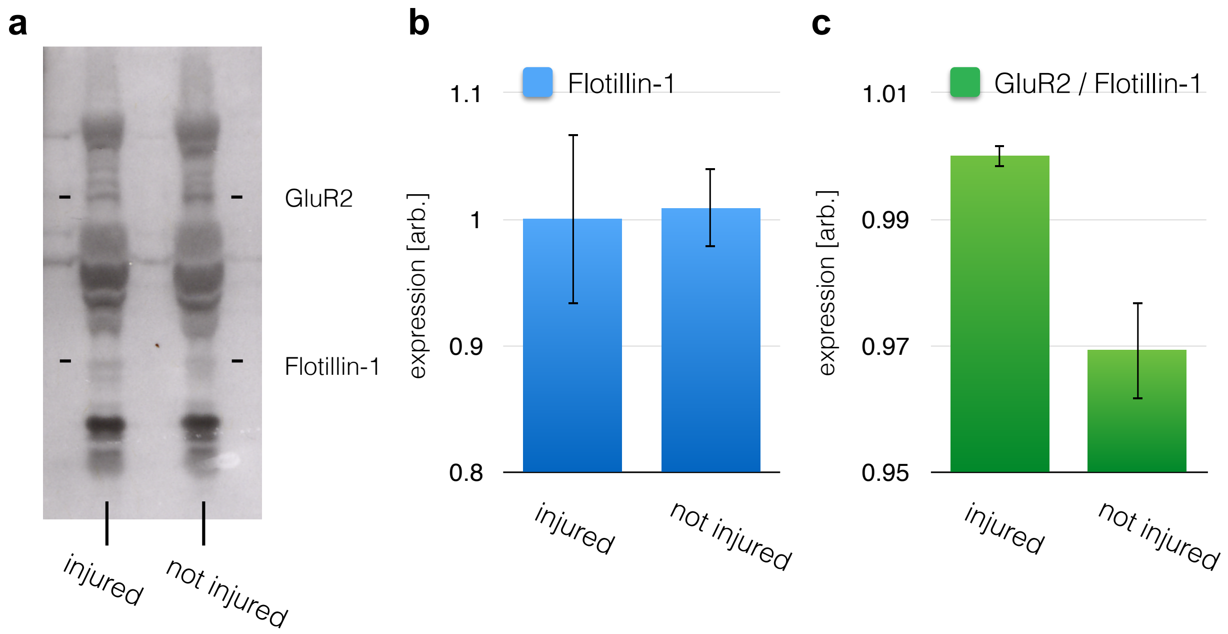

Figure S4. Measurement of GluR2+ exosomes after injury using conventional methods. a. To validate the results of μMED, exosomes were isolated using a conventional size-based method and their protein content was profiled using Western Blotting. **b.** Quantification of the Flotillin level (total exosome) for (N=6) injured and (N=6) uninjured controls, showing no significant difference. **c.** Quantification of the GluR2 level normalized by the total exosome level (Flotillin-1) for (N=6) injured and (N=6) uninjured controls, showing significant difference.

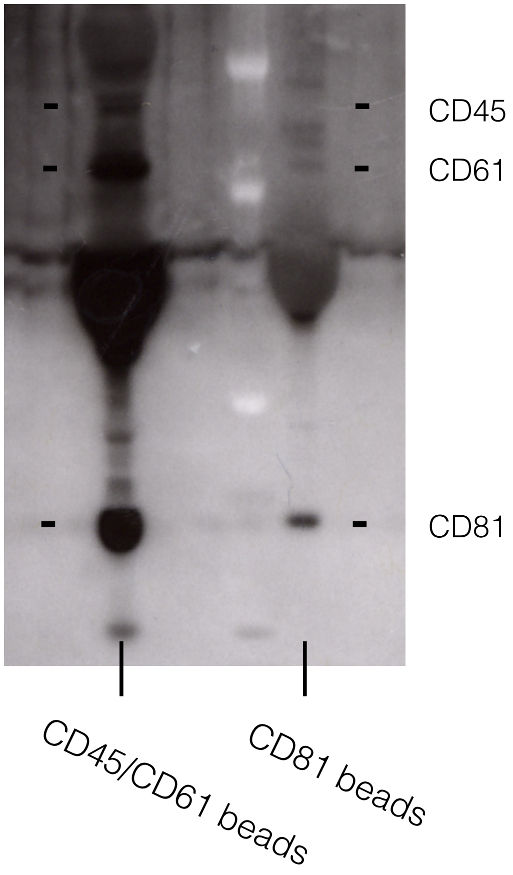

Figure S5. Western blotting on exosomes eluted from anti-CD45/CD61 beads and anti-CD81 beads. Western blotting was performed on exosomes isolated using both positive selection beads (CD81) and negative selection beads (CD45/CD61)from mouse serum. Exosomes eluted from the two bead populations were analyzed for CD45 and CD61, as well as the pan-exosome marker CD81.
